# Clinical outcomes of empirical antibiotic strategies in community-acquired pneumonia-associated sepsis: a propensity score-matched cohort study

**DOI:** 10.3389/fcimb.2026.1979459

**Published:** 2026-10-26

**Authors:** Lingshan Ding, Ruihua Hu, Yali Ying

**Affiliations:** 1 Intensive Care Unit, Zhejiang Xiaoshan Hospital, Zhejiang, China; 2 Emergency Department, Qianhai Life Guangzhou General Hospital, Guangzhou, Guangdong, China

**Keywords:** community-acquired pneumonia-associated sepsis, empirical antibiotic therapy, precision antimicrobial management, time to clinical stability, β-lactam combination therapy

## Abstract

**Background:**

Community-acquired pneumonia (CAP) complicated by sepsis remains a life-threatening infectious syndrome associated with substantial morbidity, mortality, and healthcare burden. Although β-lactam-based combination therapy is widely recommended for severe CAP, the optimal empirical antibiotic strategy for septic pneumonia remains uncertain. At the same time, empirical antibiotic selection must balance adequate early treatment against unnecessary broad-spectrum antibiotic use and the risk of antimicrobial resistance. Evidence specifically targeting this high-risk population is limited. This study aimed to compare the clinical outcomes of β-lactam monotherapy and β-lactam-based combination therapy in patients with CAP-associated sepsis.

**Methods:**

We conducted a retrospective cohort study at Zhejiang Xiaoshan Hospital from January 2021 to January 2025, including adult patients with CAP-associated sepsis who received empirical β-lactam monotherapy or β-lactam-based combination therapy with macrolides or doxycycline within 48 hours of admission. A total of 100 patients were included before propensity score matching, and 80 patients remained after 1:1 matching. The primary outcome was time to clinical stability (TTCS). Secondary outcomes included mortality, length of hospital stay, antibiotic duration, changes in SOFA score, inflammatory biomarkers, clinical cure, and adverse events. TTCS was analyzed using Kaplan–Meier analysis and Cox proportional hazards regression.

**Results:**

Median TTCS was 5.0 days with monotherapy and 4.0 days with combination therapy (P = 0.129). Combination therapy also showed favorable trends in hospitalization duration, clinical cure, inflammatory marker reduction, and organ function recovery. After adjustment for admission SOFA score, combination therapy showed a nonsignificant trend toward earlier clinical stability (adjusted HR 1.42, 95% CI 0.88–2.29; P = 0.151). No significant increase in adverse events was observed.

**Conclusions:**

In patients with CAP-associated sepsis, β-lactam-based combination therapy showed a trend toward earlier clinical stability than β-lactam monotherapy, although the difference was not statistically significant. This study addresses an important evidence gap in septic pneumonia management and supports further investigation of individualized antimicrobial strategies guided by disease severity and biological characteristics. The retrospective single-center design and relatively small sample size should be considered when interpreting these findings.

## 1 Introduction

Community-acquired pneumonia (CAP) remains one of the leading causes of infectious morbidity and mortality worldwide. Community-acquired pneumonia (CAP) is an acute pulmonary infection that develops outside the hospital or is identified shortly after hospital admission. Globally, CAP affects millions of individuals annually and imposes substantial healthcare and economic burdens. In the United States alone, CAP accounts for approximately 1.4 million emergency visits and 740,000 hospitalizations each year (Vaughn et al., 2024). The burden of CAP is particularly pronounced among older adults, who have higher rates of hospitalization and greater healthcare resource utilization (Tsoumani et al., 2023). CAP-associated sepsis refers to sepsis caused by CAP, characterized by life-threatening organ dysfunction resulting from a dysregulated host response to pulmonary infection (Vaughn et al., 2024). Despite advances in antimicrobial therapy and intensive care, pneumonia-associated sepsis remains a major challenge in modern medicine.

The cornerstone of CAP management is early empirical antibiotic therapy (Bassetti et al., 2024; Seo et al., 2024; Vaughn et al., 2024). However, the optimal empirical regimen for CAP with sepsis remains uncertain. Current guidelines recommend β-lactam-based combination therapy, particularly β-lactam plus a macrolide, for patients with severe CAP (Martin-Loeches et al., 2023). β-Lactam antibiotics provide core coverage against common bacterial pathogens in CAP and therefore form the backbone of many recommended empirical regimens. Prior antibiotic exposure may alter pathogen detection and treatment response, making it important to distinguish the effects of the initial empirical regimen. Nevertheless, recent clinical studies have questioned whether combination therapy provides additional benefits over β-lactam monotherapy (Wei et al., 2025). Most previous investigations focused on general hospitalized CAP populations rather than patients with concurrent sepsis (Bai et al., 2024). Therefore, evidence directly supporting antibiotic selection in septic pneumonia remains limited. These uncertainties highlight the need to further evaluate empirical antibiotic strategies specifically in patients with CAP-associated sepsis.

Several critical gaps remain unresolved in current research. First, previous randomized trials often excluded patients with severe organ dysfunction or sepsis (Martin-Loeches et al., 2023). Second, observational studies supporting combination therapy were frequently affected by disease severity bias and treatment selection bias (Wei et al., 2025). Third, the mechanisms underlying potential benefits of macrolides or doxycycline remain incompletely understood. Beyond antimicrobial activity, these agents may regulate excessive inflammatory responses and influence host immune balance (Zimmermann et al., 2018). However, whether these immunomodulatory effects translate into improved clinical recovery in septic pneumonia remains unclear (Al-Zergani et al., 2025). Addressing these questions is essential for developing precision antimicrobial therapy.

In this study, we aimed to provide new evidence regarding empirical antibiotic selection for CAP complicated by sepsis. Unlike previous studies, we specifically focused on this vulnerable population with high clinical complexity. We applied propensity score matching to minimize baseline differences between treatment strategies. The clinical effectiveness and safety of β-lactam monotherapy were compared with β-lactam-based combination therapy using macrolides or doxycycline. Clinical stability time was selected as the primary endpoint because it reflects both infection control and physiological recovery. Our findings provide important real-world evidence for optimizing antimicrobial management in septic pneumonia. This study may contribute to the development of individualized treatment strategies and improve outcomes for patients with severe community-acquired infections.

## 2 Methods

### 2.1 Study design

This retrospective cohort study was conducted at Zhejiang Xiaoshan Hospital between January 2021 and January 2025. Adult patients with community-acquired pneumonia (CAP) complicated by sepsis were identified from the electronic medical records of the Department of Respiratory Medicine and intensive care unit. Patients who met the eligibility criteria were included in the study. Consecutive eligible patients during the study period were included. No formal sample size calculation was performed because this retrospective study included all eligible patients available during the predefined study period. Demographic and clinical data, laboratory findings, antibiotic treatment, and clinical outcomes were collected from their medical records. The study was approved by the Ethics Committee of Zhejiang Xiaoshan Hospital (Approval No. XiaoYi Lun Shen 2026 Lun No. 037). The requirement for informed consent was waived because of the retrospective study design. The study was conducted in accordance with the Declaration of Helsinki.

### 2.2 Inclusion and exclusion criteria

Patients were eligible for inclusion if they met the following criteria: (1) aged ≥18 years; (2) diagnosed with CAP according to the Guidelines for the Diagnosis and Treatment of Adult Community-Acquired Pneumonia in China (2016), CAP was defined as an acute pulmonary infection acquired outside the hospital, with compatible clinical features and a new pulmonary infiltrate on chest imaging; (3) Sepsis was defined as suspected or confirmed infection accompanied by an acute increase in the Sequential Organ Failure Assessment (SOFA) score of ≥2 points attributable to infection; (4) hospitalized for at least 48 h; and (5) received empirical antibiotic therapy within 48 h after admission, Empirical antibiotic therapy was defined as systemic antibiotic treatment initiated before definitive microbiological results were available and within 48 h after admission. CAP and sepsis eligibility was retrospectively verified by two investigators through independent review of the medical records, laboratory findings, and chest imaging reports according to the predefined diagnostic criteria. Disagreements were resolved by discussion with a third investigator.

Patients were excluded if they met any of the following criteria: (1) received systemic antibiotic therapy for the current episode of pneumonia for >24 h within 72 h before admission; (2) had another identified source of infection associated with sepsis, such as urinary tract, intra-abdominal, or central nervous system infection, patients with multiple potential infection sources were excluded when CAP could not be established as the primary source of sepsis; (3) had significant immunosuppression, including active tuberculosis, human immunodeficiency virus infection, solid-organ or bone marrow transplantation, chemotherapy within the previous 3 months, or long-term immunosuppressive therapy; (4) had end-stage liver disease (Child–Pugh class C) or end-stage kidney disease requiring maintenance hemodialysis; (5) had a documented allergy to the antibiotics evaluated in this study; (6) were pregnant or breastfeeding; or (7) had incomplete clinical data for assessing time to clinical stability.

### 2.3 Patient grouping

Patients were classified into two groups according to the empirical antibiotic regimen initiated within 48 h after admission. The monotherapy group received a β-lactam antibiotic alone, including ceftriaxone, cefotaxime, piperacillin/tazobactam, or ceftazidime. The combination group received a β-lactam antibiotic together with a macrolide or doxycycline. Macrolide therapy included azithromycin (0.5 g/day) or clarithromycin (0.5 g twice daily), while doxycycline was administered at an initial dose of 0.2 g followed by 0.1 g twice daily. The antibiotic regimens reflected the treatments documented in routine clinical practice and were not assigned by the investigators. Two researchers independently reviewed the medical records using a standardized data collection form. Any discrepancies were resolved through review by a third researcher to ensure data accuracy.

### 2.4 Clinical outcome assessment

The primary outcome was time to clinical stability (TTCS), defined as the time from hospital admission to the first achievement of clinical stability that was maintained for at least 24 h. Clinical stability required all of the following criteria: body temperature ≤37.8 °C, heart rate ≤100 beats/min, respiratory rate ≤24 breaths/min, systolic blood pressure ≥90 mmHg, and oxygen saturation ≥90% or arterial partial pressure of oxygen ≥60 mmHg while breathing room air.

Secondary outcomes included in-hospital all-cause mortality, length of hospital stay, duration of antibiotic treatment, changes in Sequential Organ Failure Assessment (SOFA) score, changes in inflammatory biomarkers, and clinical cure. Length of hospital stay was calculated from admission to discharge or in-hospital death. Antibiotic duration was defined as the number of days from the first to the last dose of systemic antibiotic therapy. SOFA scores were assessed at admission and 72 h after treatment initiation, and the change in SOFA score (ΔSOFA) was calculated as the 72-h value minus the admission value. Changes in C-reactive protein (CRP) and procalcitonin (PCT) were assessed over the same 72-h period and expressed as percentage changes from admission values. Clinical cure was defined as complete or substantial improvement in pneumonia-related symptoms and signs without the need to change or escalate the antibiotic regimen.

### 2.5 Propensity score matching and statistical analysis

Statistical analyses were performed using R software (version 4.3.1; R Foundation for Statistical Computing, Vienna, Austria). Continuous variables were assessed for normality using the Shapiro–Wilk test. Normally distributed variables are presented as mean ± standard deviation and were compared using the independent-samples t-test, whereas non-normally distributed variables are presented as median (interquartile range) and were compared using the Mann–Whitney U test. Categorical variables are presented as number (percentage) and were compared using the chi-square test or Fisher’s exact test, as appropriate. All tests were two-sided, with P<0.05 considered statistically significant. No imputation was performed for missing data; analyses were based on available complete observations.

Propensity score matching (PSM) was used to reduce treatment-selection bias and baseline confounding between the two treatment groups. Propensity scores were estimated using logistic regression based on age, sex, Charlson Comorbidity Index, admission SOFA score, CURB-65 score, and vasopressor use. Patients were matched 1:1 using nearest-neighbor matching with a caliper width of 0.2 standard deviations of the propensity score. Covariate balance was assessed using standardized mean differences (SMDs), with an absolute SMD <0.10 indicating adequate balance. The matched cohort was used for the primary efficacy analyses.

Time to clinical stability (TTCS) was analyzed using the Kaplan–Meier method and compared between groups using the log-rank test. Cox proportional hazards regression was used to estimate hazard ratios (HRs) and 95% confidence intervals (CIs). Admission SOFA score was included in the adjusted model to account for residual differences in disease severity. The proportional hazards assumption was assessed using Schoenfeld residuals.

Secondary outcomes were analyzed in the matched cohort. Because of the small number of in-hospital deaths, mortality was compared using Fisher’s exact test without regression modeling. Safety analyses were performed in the full cohort before propensity score matching. To explore whether the association between empirical antibiotic strategy and time to clinical stability differed across clinically relevant patient characteristics, exploratory heterogeneity analyses were performed. Exploratory subgroup analyses were performed according to age (<65 vs. ≥65 years) and admission SOFA score (<6 vs. ≥6). Cox proportional hazards models were used to estimate the association between treatment strategy and TTCS within each subgroup. Cox proportional hazards models incorporating treatment-by-subgroup interaction terms were used to assess potential effect modification. Effect estimates were reported as HRs for TTCS, mean or median differences for continuous outcomes, and risk differences for binary outcomes, with 95% CIs where applicable. Etiological distributions between the treatment groups were compared using the Fisher–Freeman–Halton exact test. We then performed additional analyses to examine whether atypical pathogen detection influenced the association between antibiotic strategy and clinical stability. These analyses included adjustment for atypical pathogen detection, exclusion of patients with detected atypical pathogens, Cox regression stratified by atypical pathogen status, and assessment of the interaction between treatment strategy and atypical pathogen detection. All pathogen-specific analyses were considered exploratory.

### 2.6 Microbiological assessment

Microbiological test results were obtained from the medical records. Tests were performed according to clinical judgment and included cultures of sputum or endotracheal aspirates and blood cultures. Testing for atypical pathogens included Legionella urinary antigen and serology or PCR for Mycoplasma pneumoniae and Chlamydia pneumoniae. Respiratory viruses were assessed using multiplex PCR. In selected patients, targeted or metagenomic next-generation sequencing (tNGS/mNGS) of bronchoalveolar lavage fluid was also performed. Antimicrobial susceptibility results were interpreted according to CLSI M100 breakpoints. Based on the available microbiological results, patients were classified into five mutually exclusive groups: typical bacteria, atypical pathogens, respiratory viruses, bacterial–viral co-detection, or no pathogen identified.

## 3 Results

### 3.1 Patient selection, propensity score matching, and clinical characteristics

During the study period from January 2021 to January 2025, 238 adult patients with community-acquired pneumonia complicated by sepsis were identified through the electronic medical record system. After screening according to the predefined inclusion and exclusion criteria, 100 patients were eligible for inclusion in the study (**Figure 1**). Among them, 50 patients received β-lactam monotherapy, whereas 50 patients received β-lactam-based combination therapy with macrolides or doxycycline. Before propensity score matching, baseline characteristics and disease severity indicators were compared between the two treatment groups (**Table 1**). The two groups showed generally comparable demographic characteristics and comorbidity profiles, including age, sex, body mass index, hypertension, diabetes, chronic obstructive pulmonary disease, coronary heart disease, chronic kidney disease, and cerebrovascular disease. However, because treatment selection in routine clinical practice may be influenced by disease severity and clinical conditions, potential differences in baseline severity indicators, such as SOFA score, CURB-65 score, intensive care unit admission, and inflammatory markers, were considered potential sources of confounding.

**Figure 1:**
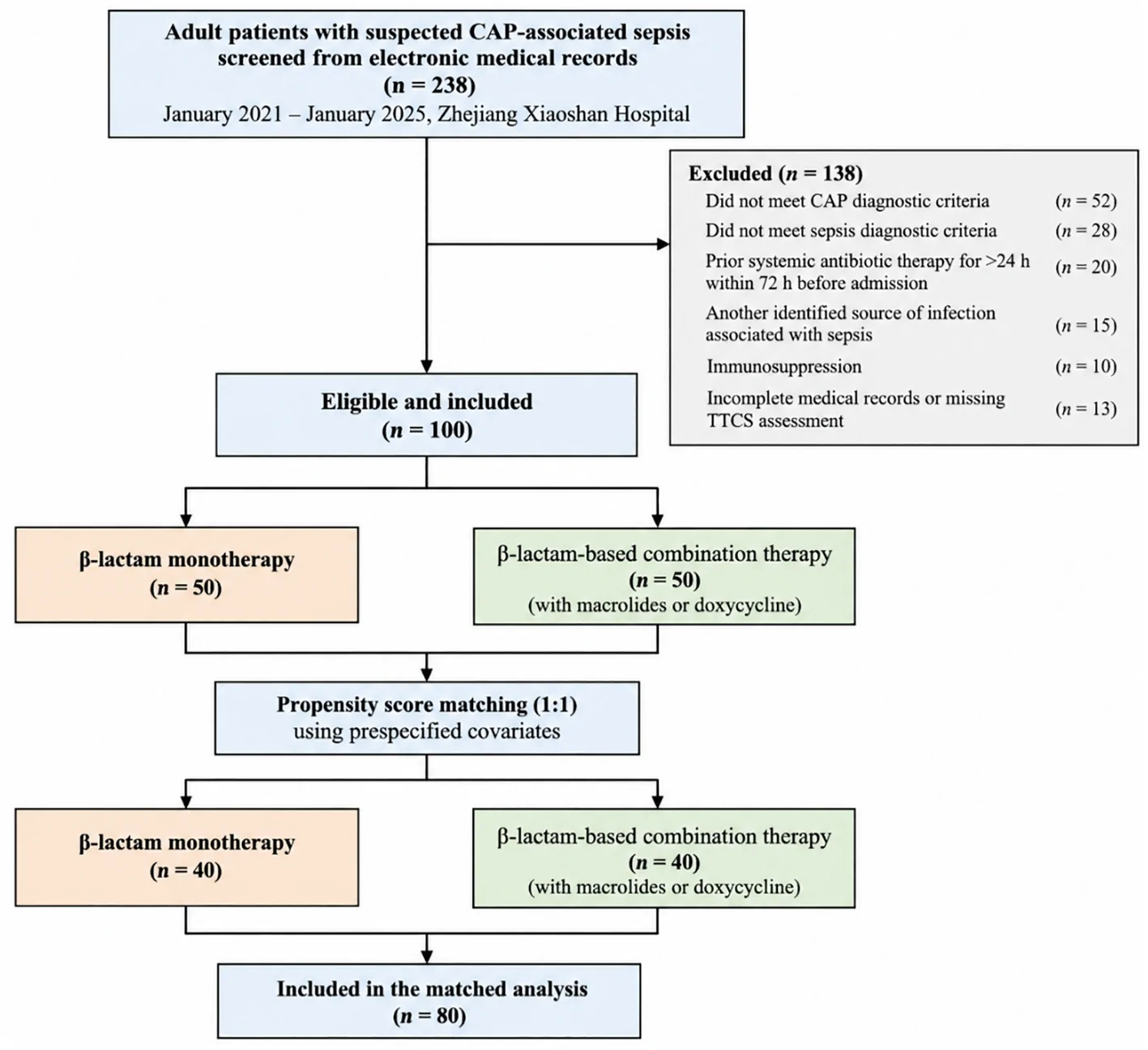
Participant selection and propensity score matching flowchart. The flowchart shows patient screening, eligibility assessment, treatment-group allocation, 1:1 propensity score matching, and inclusion in the final matched analysis. CAP, community-acquired pneumonia; PSM, propensity score matching; TTCS, time to clinical stability.

**Table 1:** Clinical characteristics of patients before propensity score matching (n=100).

Characteristic	Monotherapy (n = 50)	Combination (n = 50)	Statistic (t/χ²)	P-value
Age (years)	63.55 ± 14.62	61.72 ± 15.18	t = 0.619	0.538
Male, n (%)	31 (62.00)	34 (68.00)	χ² = 0.396	0.529
BMI (kg/m²)	24.38 ± 3.85	25.02 ± 4.15	t = −0.796	0.428
Hypertension, n (%)	26 (52.00)	22 (44.00)	χ² = 0.641	0.423
Diabetes mellitus, n (%)	17 (34.00)	20 (40.00)	χ² = 0.386	0.534
COPD, n (%)	13 (26.00)	15 (30.00)	χ² = 0.198	0.656
Coronary heart disease, n (%)	10 (20.00)	9 (18.00)	χ² = 0.065	0.799
CKD, n (%)	6 (12.00)	7 (14.00)	χ² = 0.088	0.766
Cerebrovascular disease, n (%)	7 (14.00)	8 (16.00)	χ² = 0.078	0.78
Admission SOFA, median [IQR]	5.0 [3.0, 7.0]	6.0 [4.0, 8.0]	U = 1108.5	0.329
CURB-65, median [IQR]	2.0 [1.0, 3.0]	2.0 [2.0, 3.0]	U = 1137.0	0.436
SOFA ≥ 2, n (%)	17 (34.00)	23 (46.00)	χ² = 1.500	0.221
ICU admission, n (%)	11 (22.00)	16 (32.00)	χ² = 1.268	0.26
WBC (×10^9^/L)	13.60 ± 5.12	14.28 ± 5.58	t = −0.640	0.524
CRP (mg/L), median [IQR]	130.5 [74.2, 201.1]	141.8 [84.5, 225.3]	U = 1132.5	0.417
PCT (ng/mL), median [IQR]	2.15 [0.62, 5.91]	2.88 [0.79, 7.35]	U = 1158.0	0.526
Creatinine (μmol/L)	87.60 ± 32.35	91.50 ± 36.22	t = −0.571	0.569
Albumin (g/L)	32.22 ± 5.60	31.30 ± 6.09	t = 0.784	0.435

To reduce potential confounding caused by differences in patient characteristics between the two treatment groups, propensity score matching was performed using age, sex, Charlson Comorbidity Index, admission SOFA score, CURB-65 score, and vasopressor use as matching variables. A 1:1 nearest-neighbor matching approach was applied, resulting in 40 matched pairs and a final matched cohort of 80 patients. After matching, all included covariates achieved adequate balance, with absolute standardized mean differences below 0.10 (**Figure 2** and **Table 2**), supporting the comparability of the two groups for subsequent outcome analyses.

**Figure 2:**
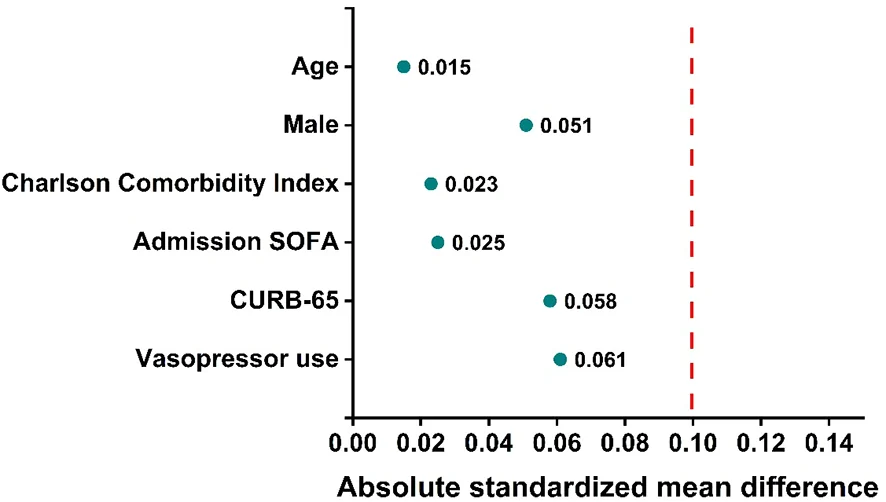
Standardized mean differences in covariates after propensity score matching.

**Table 2:** Comparison of baseline data after propensity score matching (n=80).

Characteristic	Monotherapy (n = 40)	Combination (n = 40)	SMD
Age (years)	62.88 ± 14.69	63.10 ± 15.12	0.015
Male, n (%)	25 (62.50)	24 (60.00)	0.051
Charlson Comorbidity Index	1.85 ± 1.33	1.82 ± 1.29	0.023
Admission SOFA, median [IQR]	5.0 [3.0, 7.0]	5.0 [4.0, 8.0]	0.025
CURB-65, median [IQR]	2.0 [2.0, 3.0]	2.0 [2.0, 3.0]	0.058
Vasopressor use, n (%)	8 (20.00)	9 (22.50)	0.061

### 3.2 Effect of empirical antibiotic strategy on time to clinical stability

The primary outcome of this study was time to clinical stability (TTCS), which reflects the overall recovery process after initial antibiotic treatment. In the propensity score–matched cohort of 80 patients, patients receiving β-lactam-based combination therapy showed a numerically shorter time to clinical stability than those receiving β-lactam monotherapy. The median TTCS was reduced from 5.00 days in the monotherapy group to 4.00 days in the combination therapy group, corresponding to an earlier recovery pattern during hospitalization. Consistently, the mean TTCS was shorter in patients receiving combination therapy (4.72 ± 1.83 days) compared with monotherapy (5.48 ± 1.89 days). Kaplan–Meier analysis demonstrated a progressive separation of clinical recovery curves favoring combination therapy (**Figure 3** and **Table 3**). These findings showed a numerical trend toward earlier clinical stabilization with β-lactam-based combination therapy, although the between-group difference was not statistically significant.

**Figure 3:**
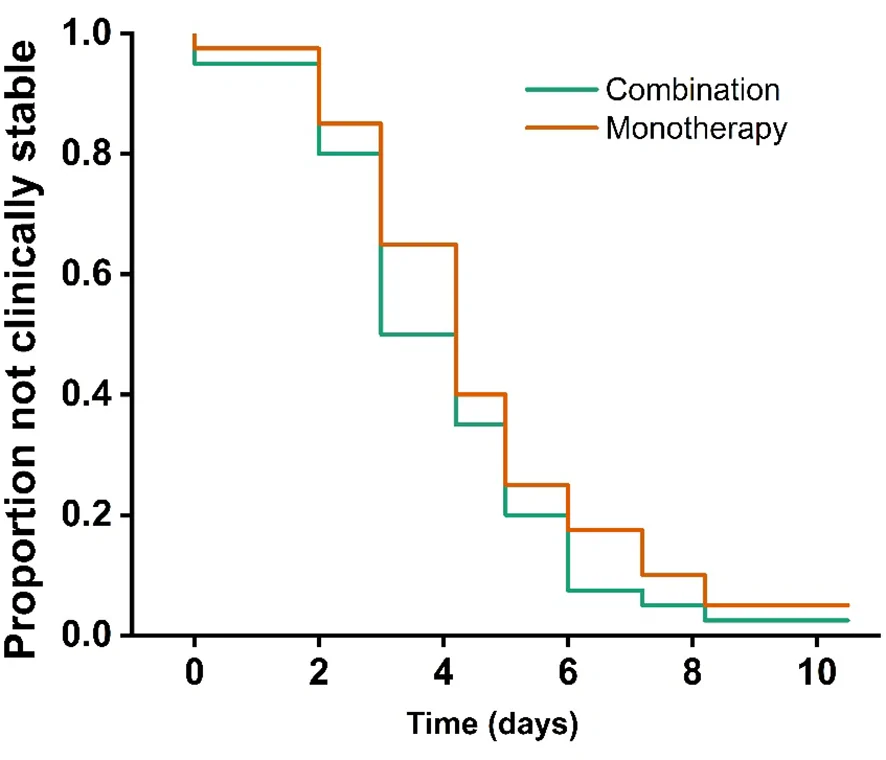
Kaplan–Meier curves for time to clinical stability according to empirical antibiotic strategy.

**Table 3:** Main outcome: Comparison of clinical stability time (PSM cohort) n=80.

Outcome	Monotherapy (n = 40)	Combination (n = 40)	Statistic	P-value
Median TTCS (days, 95% CI)	5.00 (4.31, 5.69)	4.00 (3.35, 4.65)	χ² = 2.305	0.129
Mean TTCS (days, Mean ± SD)	5.48 ± 1.89	4.72 ± 1.83	t = 1.828	0.071
7-day clinical stability, n (%)	33 (82.50)	37 (92.50)	χ² = 1.829	0.176

### 3.3 Clinical recovery and safety outcomes after empirical antibiotic therapy

We further assessed secondary clinical outcomes to characterize the overall recovery profile and safety of different empirical antibiotic strategies (**Table 4**). Compared with β-lactam monotherapy, the combination therapy group showed a numerically shorter median hospital stay (8.0 vs. 10.0 days) and a lower overall antibiotic exposure (8.75 ± 3.42 vs. 9.70 ± 3.79 days). The proportion of patients achieving clinical cure was higher in the combination group (92.5% vs. 85.0%). Numerically greater early improvements in inflammatory and organ dysfunction markers were also observed in the combination therapy group, including larger reductions in CRP and procalcitonin levels and a more favorable change in SOFA score within 72 h. However, none of these between-group differences reached statistical significance (**Figures 4A–C**). Safety analysis revealed comparable adverse event profiles between groups, supporting the tolerability of the combination approach.

**Table 4:** Comparison of secondary clinical outcomes between treatment groups after propensity score matching (n=80).

Outcome	Monotherapy (n = 40)	Combination (n = 40)	Statistic	P-value
In-hospital mortality, n (%)	2 (5.00)	1 (2.50)	—	1.000
Total hospital stay (days), median [IQR]	10.0 [7.0, 14.3]	8.0 [6.0, 11.3]	U = 648.0	0.144
Antibiotic duration (days)	9.70 ± 3.79	8.75 ± 3.42	t = 1.172	0.245
Clinical cure rate, n (%)	34 (85.00)	37 (92.50)	—	0.314
72h ΔSOFA	−1.18 ± 1.49	−1.62 ± 1.74	t = 1.219	0.227
72h CRP change rate (%), median [IQR]	−47.1 [−61.5, −30.3]	−56.5 [−71.2, −37.4]	U = 639.5	0.106
72h PCT change rate (%), median [IQR]	−51.2 [−67.6, −33.8]	−60.9 [−76.1, −41.5]	U = 635.0	0.098

**Figure 4:**
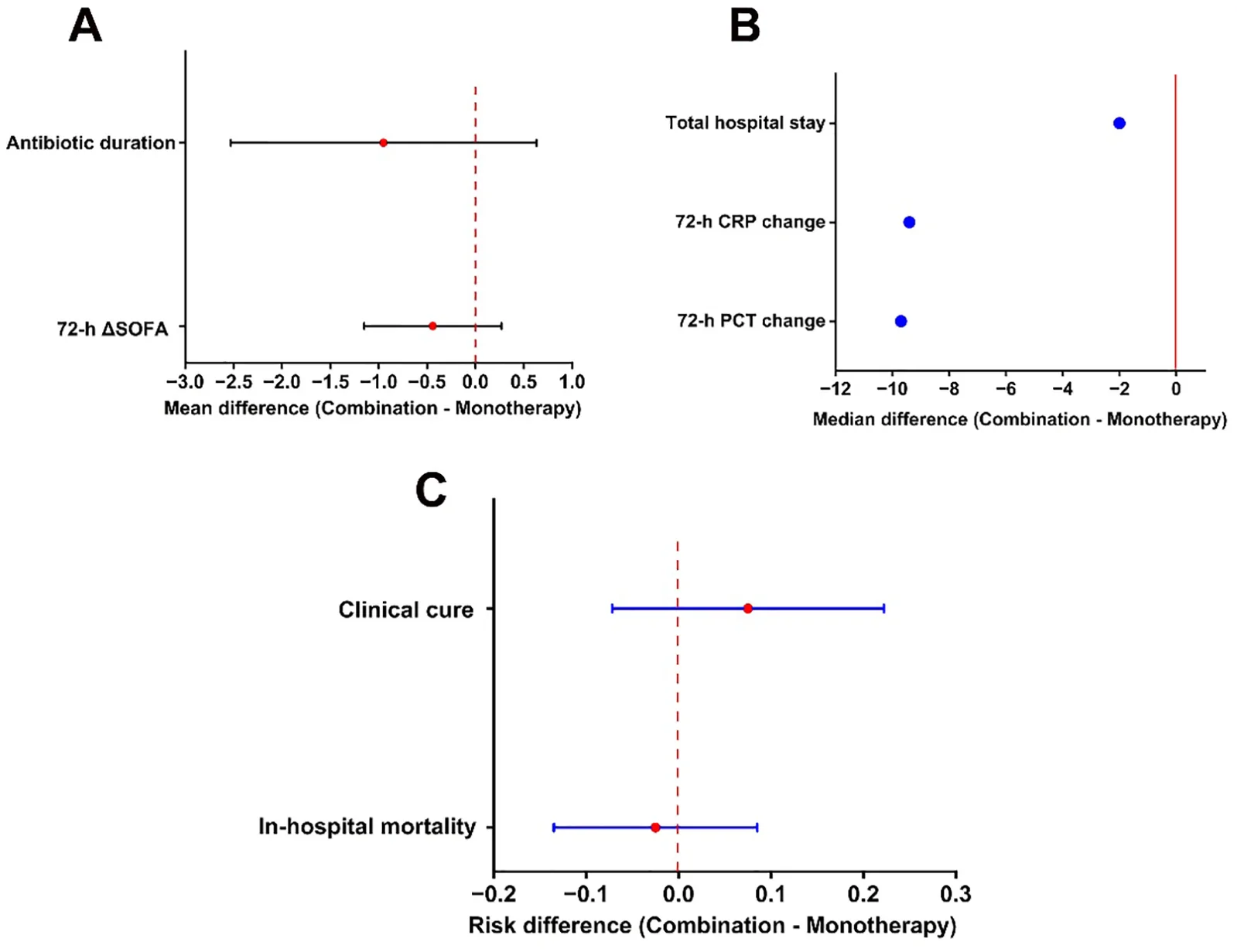
Comparison of secondary clinical outcomes between empirical antibiotic strategies after propensity score matching. **(a)** Mean differences for continuous outcomes, including antibiotic duration and changes in SOFA score. **(b)** Median differences for non-normally distributed outcomes, including length of hospital stay and changes in inflammatory biomarkers (CRP and procalcitonin). **(c)** Risk differences for binary outcomes, including in-hospital mortality and clinical cure rate.

### 3.4 Factors associated with time to clinical stability

To explore potential determinants of clinical recovery, univariable Cox regression analyses were performed in the propensity score–matched cohort. The analysis examined the associations of empirical antibiotic strategy and baseline disease severity with TTCS, (**Table 5**). Patients receiving β-lactam-based combination therapy showed a higher probability of reaching clinical stability compared with those receiving β-lactam monotherapy (HR 1.39, 95% CI 0.86–2.24). In contrast, increasing admission SOFA scores were associated with a reduced likelihood of early stabilization (HR 0.94 per point increase, 95% CI 0.87–1.01), suggesting that initial organ dysfunction may delay recovery. These exploratory findings indicate that both treatment selection and baseline physiological status may contribute to the variation in clinical recovery among patients with pneumonia-associated sepsis.

**Table 5:** Single factor Cox regression analysis: factors influencing clinical stability time (PSM cohort, n=80).

Variable	Unadjusted HR (95% CI)	P-value
Combination vs. Monotherapy	1.39 (0.86, 2.24)	0.178
Admission SOFA (per 1-point increase)	0.94 (0.87, 1.01)	0.104

### 3.5 Multivariable Cox regression analysis of time to clinical stability

A multivariable Cox regression model was subsequently established to determine whether empirical antibiotic strategy was independently associated with clinical stabilization after accounting for baseline disease severity. After adjustment for admission SOFA score, β-lactam-based combination therapy showed a nonsignificant trend toward a higher likelihood of achieving clinical stability (adjusted HR 1.42, 95% CI 0.88–2.29; P = 0.151, **Table 6**). Higher admission SOFA scores also showed a nonsignificant trend toward delayed clinical stabilization (adjusted HR 0.92 per point increase, 95% CI 0.85–1.02; P = 0.073). Together, these adjusted analyses showed numerical associations of treatment strategy and baseline disease severity with TTCS, although neither association reached statistical significance.

**Table 6:** Multivariate Cox regression analysis: factors influencing clinical stability time (PSM cohort).

Variable	Adjusted HR (95% CI)	P-value
Combination vs. Monotherapy	1.42 (0.88, 2.29)	0.151
Admission SOFA (per 1-point increase)	0.92 (0.85, 1.02)	0.073

### 3.6 Exploratory analysis of treatment effects across clinical subgroups

To explore whether the association between empirical antibiotic strategy and clinical recovery varied according to patient age or baseline disease severity, exploratory subgroup analyses were performed according to age and admission SOFA score (**Figure 5**). Across the evaluated age and SOFA subgroups, the point estimates generally favored β-lactam-based combination therapy, although the confidence intervals were wide and crossed 1.0. The association appeared numerically stronger among patients aged ≥65 years and those with admission SOFA scores ≥6. However, given the small subgroup sizes and statistical uncertainty, these findings should be considered exploratory and do not establish differential treatment effects across patient subgroups.

**Figure 5:**
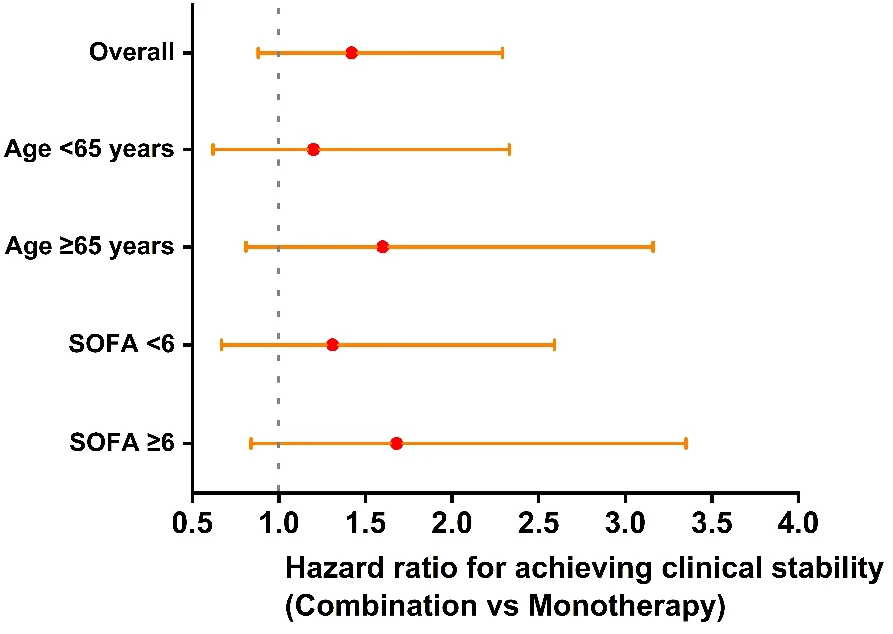
Exploratory subgroup analysis of the association between empirical antibiotic strategy and time to clinical stability according to age and admission SOFA score. Hazard ratios >1 favor β-lactam-based combination therapy. Error bars indicate 95% confidence intervals.

### 3.7 Safety outcomes associated with empirical antibiotic strategies

Safety outcomes were evaluated in the full cohort before propensity score matching to capture all documented adverse events during hospitalization. The incidence of adverse events was compared between patients receiving β-lactam monotherapy and those receiving β-lactam-based combination therapy (**Table 7** and **Figure 6**).

**Table 7:** Comparison of safety outcomes in the full cohort (n=100).

Adverse event	Monotherapy, n (%)	Combination, n (%)	P-value
Any adverse event	14 (28.00)	19 (38.00)	0.288
Grade ≥3 adverse events	1 (2.00)	2 (4.00)	1.000
Elevated liver enzymes	3 (6.00)	7 (14.00)	0.182
Gastrointestinal reactions	6 (12.00)	9 (18.00)	0.401
Rash/allergy	1 (2.00)	2 (4.00)	1.000
Nephrotoxicity	2 (4.00)	1 (2.00)	1.000
Discontinuation due to adverse events	1 (2.00)	3 (6.00)	0.618

**Figure 6:**
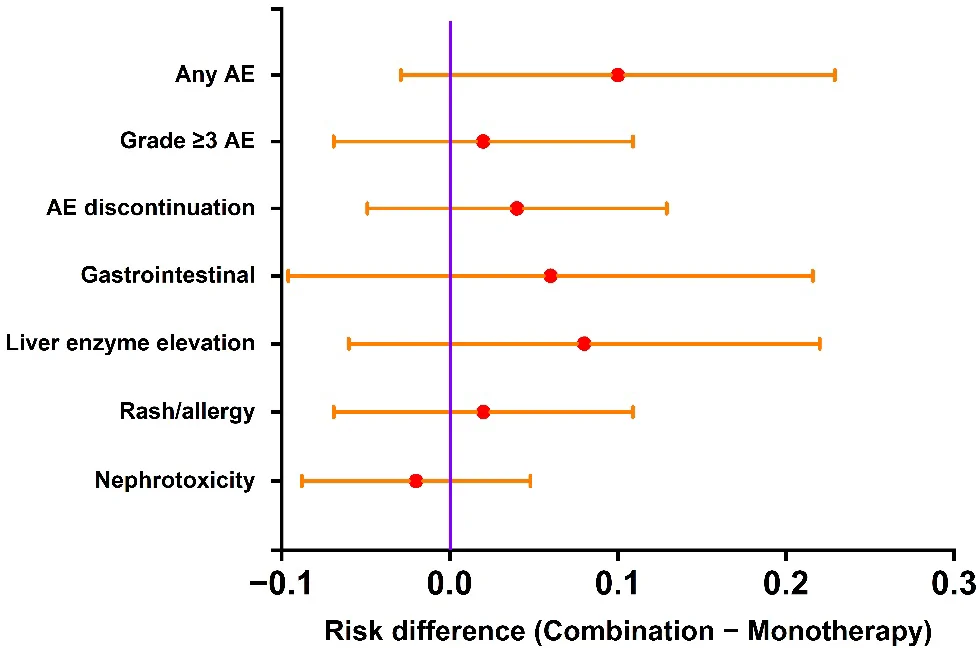
Comparison of adverse events between empirical antibiotic strategies.

The overall incidence of adverse events was low in both treatment groups, and no significant differences were observed in the occurrence of rash, gastrointestinal symptoms, liver function abnormalities, renal function abnormalities between the two groups (all P>0.05). These findings suggest that β-lactam-based combination therapy did not show an increased risk of commonly recorded adverse events compared with monotherapy in this cohort.

### 3.8 Microbiological characteristics and pathogen-stratified analysis

Microbiological testing identified at least one pathogen in 45 of the 80 matched patients (56.3%). Pathogen detection rates were similar between the monotherapy and combination groups (55.0% vs. 57.5%; P = 1.000). The overall etiological distribution was also comparable between groups (P = 0.92; **Figure 7A**). Typical bacteria were identified in 27.5% and 22.5% of patients, respectively, whereas atypical pathogens were detected in 10.0% and 17.5% (P = 0.52). Among isolates with available susceptibility testing, the initial β-lactam was active *in vitro* against 17 of 19 tested isolates. Erythromycin resistance was detected in 6 of 7 S. pneumoniae isolates. Because atypical pathogens were numerically more frequent in the combination group, additional analyses were performed to assess their potential influence on the primary outcome. The association between combination therapy and earlier clinical stability remained similar after adjustment for atypical pathogen detection (HR 1.37, 95% CI 0.84–2.23) and after excluding patients with detected atypical pathogens (HR 1.33, 95% CI 0.79–2.24). No evidence of an interaction between treatment strategy and atypical pathogen detection was observed (P for interaction = 0.64; **Figure 7B**). These exploratory analyses therefore showed no clear evidence that atypical pathogen detection materially altered the association between treatment strategy and TTCS.

**Figure 7:**
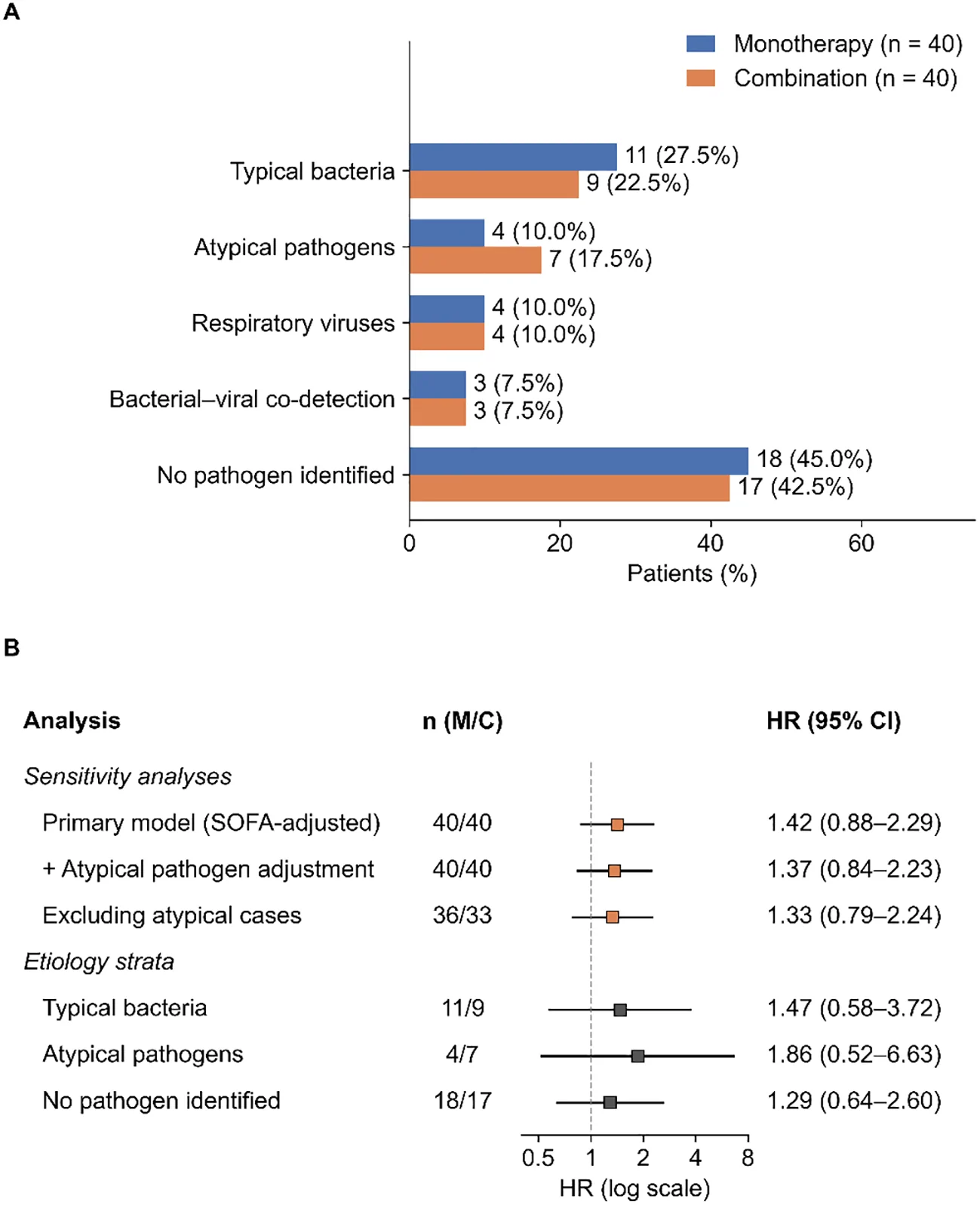
Microbiological etiology and pathogen-stratified analysis in the matched cohort (n = 80). **(A)** Etiological distribution by treatment group. **(B)** SOFA-adjusted HRs for clinical stability; HR > 1 favors combination therapy.

## 4 Discussion

### 4.1 Major findings and clinical significance

In this retrospective cohort study, we evaluated the comparative effectiveness of β-lactam monotherapy and β-lactam-based combination therapy with macrolides or doxycycline in hospitalized patients with community-acquired pneumonia complicated by sepsis. By applying propensity score matching to minimize baseline differences, our study provides real-world evidence regarding an important but unresolved clinical question: whether expanded empirical antimicrobial coverage can improve early recovery in patients with septic pneumonia. Our findings showed numerical trends toward earlier clinical stability and shorter hospitalization with combination therapy, although these between-group differences were not statistically significant. Together, these results provide preliminary real-world evidence for further evaluation of empirical antibiotic strategies in this high-risk population. In patients with pneumonia-associated sepsis, where rapid infection control and early physiological recovery are closely linked to prognosis, optimizing empirical antimicrobial strategies remains of critical importance (Khilnani et al., 2024). Unlike many previous studies conducted in broader hospitalized CAP populations, our study specifically focused on patients with concomitant sepsis, thereby addressing an important evidence gap (Wei et al., 2025).

### 4.2 Contribution to current understanding of antibiotic strategies in septic pneumonia

The optimal empirical antibiotic strategy for CAP with sepsis remains controversial. β-lactam antibiotics provide effective coverage against major typical pathogens. However, septic pneumonia represents a complex disease state characterized by diverse microbial profiles, high inflammatory burden, and dysregulated host immune responses (Cavallazzi and Ramirez, 2024; Li et al., 2024; Pickens et al., 2024). Therefore, antibiotic selection in this population should consider not only pathogen eradication but also the potential influence on inflammatory regulation and disease progression.

Previous studies have provided valuable but incomplete evidence. For example, a randomized clinical trial by Garin et al. in hospitalized patients with moderately severe CAP failed to demonstrate the noninferiority of β-lactam monotherapy compared with β-lactam plus macrolide therapy (Garin et al., 2014). However, these patients were not specifically characterized by sepsis, limiting direct extrapolation to critically ill populations. Similarly, a large observational cohort study reported that β-lactam plus macrolide therapy was associated with reduced mortality among patients with pneumococcal CAP and a strong inflammatory response (Ceccato et al., 2019; Niederman and Torres, 2022). These findings suggest that the potential benefits of combination therapy may be restricted to specific biological phenotypes rather than universally applicable to all patients. Our findings showed numerical trends toward earlier clinical stability and shorter hospitalization with combination therapy, although these between-group differences were not statistically significant. These findings suggest a potential clinical advantage of combination therapy that requires further evaluation in larger prospective cohorts.

### 4.3 Biological rationale supporting combination therapy

The potential advantage of combination therapy may arise from complementary mechanisms. In addition to improving coverage against atypical pathogens, macrolides possess immunomodulatory properties that may attenuate excessive inflammatory activation (Pollock and Chalmers, 2021; Blondeau, 2022). In sepsis, uncontrolled inflammatory responses contribute significantly to endothelial injury, tissue damage, and organ dysfunction (Girardis et al., 2024). Therefore, therapies capable of simultaneously controlling microbial infection and modulating host inflammation may provide additional clinical benefits (Bode et al., 2023). Consistent with this hypothesis, our study observed more favorable trends in inflammatory biomarker improvement and organ recovery parameters among patients receiving combination therapy. Accounting for atypical pathogen detection through adjustment or exclusion produced similar estimates, suggesting that atypical pathogen distribution alone may not fully explain the observed trend. The high frequency of macrolide resistance and preserved β-lactam activity against most tested isolates provide additional context for interpreting these findings. However, the small number of patients within individual pathogen strata limits the precision of these analyses, and the relative contributions of pathogen coverage, antimicrobial susceptibility, and other potential mechanisms remain uncertain. Further mechanistic studies incorporating pathogen-specific data are needed to clarify the relative contributions of these potential mechanisms.

### 4.4 Implications for precision antimicrobial therapy

An important implication of our findings is that combination therapy may not be necessary for all CAP patients but may provide greater value in selected high-risk individuals. Exploratory analyses showed a numerically stronger association with earlier clinical stability among patients with higher disease severity, although the subgroup estimates were imprecise. This observation aligns with previous reports showing that the advantages of β-lactam/macrolide therapy are more evident among severe CAP populations (Martin-Loeches et al., 2023; Wang et al., 2025). For instance, studies conducted in intensive care settings have reported improved outcomes with β-lactam/macrolide combinations among critically ill CAP patients compared with β-lactam-based regimens without macrolides (Martin-Loeches et al., 2023). These findings suggest that future studies should further evaluate whether patient stratification can help identify individuals who may benefit from combination therapy rather than assuming a uniform benefit across all patients. Integrating clinical severity scores, inflammatory biomarkers, and microbiological characteristics may help identify patients who are most likely to benefit from additional antimicrobial agents.

### 4.5 Limitations and future perspectives

Several limitations should be acknowledged. First, the retrospective single-center design may have introduced selection bias. Although propensity score matching improved the balance of measured baseline characteristics, it cannot account for unmeasured or incompletely measured confounders, and residual confounding may therefore remain. Second, the relatively small sample size may have limited the ability to fully capture treatment effects, particularly among specific severity subgroups. Third, the combination therapy group included both macrolides and doxycycline, and their distinct antimicrobial and immunomodulatory effects could not be separately evaluated. Fourth, microbiological testing was not protocolized, some specimens were collected after antibiotic initiation, and 43.8% of patients had no identified pathogen. These factors limit pathogen-specific inferences.

## 5 Conclusion

In patients with community-acquired pneumonia complicated by sepsis, β-lactam-based combination therapy with a macrolide or doxycycline showed a trend toward earlier clinical stabilization and improved recovery, without major safety concerns. These findings support further evaluation of individualized antibiotic selection for selected high-risk patients. However, large prospective studies are needed to confirm these associations and identify patients most likely to benefit. Future studies should integrate pathogen data, inflammatory biomarkers, and clinical severity measures to enable more precise antibiotic treatment.

## Data Availability

The raw data supporting the conclusions of this article will be made available by the authors, without undue reservation.
